# Novel compound heterozygous mutations in the desert hedgehog (*DHH*) gene in cases of siblings with 46,XY disorders of sexual development

**DOI:** 10.1186/s12920-022-01334-5

**Published:** 2022-08-15

**Authors:** Jia Wei, Jiaqi Wu, Wei Ru, Guangjie Chen, Lei Gao, Daxing Tang

**Affiliations:** 1 Department of Urology, Children’s Hospital, Zhejiang University School of Medicine, Hangzhou, China; 2 Department of Gastroenterology, The Affiliated Hospital of Hangzhou Normal University, Hangzhou, China

**Keywords:** Disorders of sex development, Desert hedgehog (DHH), Mutation

## Abstract

**Background:**

Disorders of sex development (DSD) are congenital disorders in which the development of the chromosomal, gonadal, or anatomical sex is atypical. Mutations in various genes can impede gonadal development, hormone synthesis, or hormone function and cause DSD.

**Methods:**

Exome sequencing was performed for two siblings with 46,XY DSD. All mutations identified by exome sequencing were confirmed by Sanger sequencing.

**Results:**

The 13-month-old younger sibling had a female appearance of the external genital with a clitoris that was assessed as Prader III and scored 2 in the external masculinization score evaluative test. The 16-year-old elder sibling had severe hypospadias. Exome sequencing revealed compound heterozygous mutations in exon 3 of *DHH* in the siblings with 46,XY DSD. The frameshift mutation (NM_021044.3: c.602delC) was derived from the father and was predicted to be deleterious. The (c.937G > T) substitution mutation was derived from the mother.

**Conclusions:**

Novel compound heterozygous mutations of *DHH* led to 46,XY DSD in two siblings. This study expands the phenotypic mutation spectra of *DHH* in patients with 46,XY DSD.

## Background

Disorders of sex development (DSD) are congenital disorders in which the development of the chromosomal, gonadal, or anatomical sex is atypical. DSD includes a wide range of phenotypes, such as 46,XY DSD, 46,XX DSD, and chromosomal DSD [1]. The incidence rate among subjects with 46,XY to have a DSD has been estimated to be 1 in 20,000 births globally [2]. For the individuals with 46,XY DSD, defects in the development of the male gonads and/or external genitalia occur.

Male sex determination in humans depends on the *SRY* gene, which is located on the Y chromosome. It also depends on the presence of several other genes located on autosomal and X-linked loci that are involved in the testis-determining pathways [3], including *SOX9*, *MAP3K1*, *NR5A1*, *WT1*, *ARTX*, and *DHH*.

*DHH,* encoding desert hedgehog (DHH), belongs to the hedgehog gene family. This gene encodes signalling molecules that play an essential role in cell proliferation, differentiation, tissue patterning, and regulation morphogenesis [4]. *DHH* is located on 12q12-q13.1. It is composed of three exons and encodes a protein of 396 amino acid residues [5]. *DHH* mRNA and protein are explicitly expressed in Sertoli cells and Schwann cells along peripheral nerves [6]. It is one of the first indications of male-specific development before overt sexual dimorphism of the gonads. Moreover, it promotes foetal Leydig cell development and the consequent formation of testis cords by activating Hedgehog signalling [7]. In humans, mutations in *DHH* have been reported in patients presenting with 46,XY DSD and with/without minifascicular polyneuropathy [5, 8–10]. Interestingly, previous literature has reported that these patients always have homozygous *DHH* mutations or compound heterozygote mutations [5, 9, 11, 12].

This study reports the genetic and molecular analysis of two siblings with 46,XY DSD who had a novel double heterozygous variant in *DHH*.

## Methods

### Study participants

This study was reviewed and approved by the Institute’s Human Research Committee of the Children’s Hospital of Zhejiang University School of Medicine. The patients were evaluated by a multidisciplinary team in the hospital, including paediatric urologists, paediatric endocrinologists, and geneticists. The parents of the siblings approved the usage of blood samples for the study.

### Genetic analysis

Genomic DNA from the proband was extracted using a QIAamp Blood Midi Kit (QIAGEN, Valencia, CA). To identify disease-causing gene variants, a GenCap panel with 168 genes associated with DSD was customized, and a capture strategy was performed using the GenCap custom enrichment kit (MyGenostics Inc, Beijing, China). An Illumina HiSeq 4000 sequencer (Illumina, San Diego, CA, USA) was used with 150 bp paired-end reads.

### Data analysis

Following sequencing, the raw data were saved as a FASTQ format. Both MGI sequencing adapters and low quality reads (< 80 bp) were filtered by cutadaptor software (http://code.google.com/p/cutadapt/). The clean reads were mapped to the UCSC hg19 human reference genome using the parameter BWA of Sentieon software.(https://www.sentieon.com/).The duplicated reads were removed using the parameter driver of Sentieon software, and the parameter driver is used to correct the base, so that the quality value of the base in the reads of the final output BAM file can be closer to the real probability of mismatch with the reference genome, and the mapped reads were used for the detection of variation. The variants of SNP and InDel were detected by the parameter driver of Sentieon software. Then, the data would be transformed to VCF format. Variants were further annotated by ANNOVAR software (http://annovar.openbioinformatics.org/en/latest/), and associated with multiple databases, such as, 1000 genome, ESP6500, dbSNP, EXAC, Inhouse (MyGenostics), HGMD, and also predicted by SIFT, PolyPhen-2, MutationTaster, GERP +  + . The pathogenicity of variation loci was also analyzed according to ACMG (American College of Medical Genetics and Genomics) genetic variation classification criteria and guidelines.

### Validation by sanger sequencing

All mutations identified by HiSeq 4000 sequencing were confirmed by Sanger sequencing. Genomic DNA from all available family members were obtained for Sanger sequencing. A pair of primers were designed to amplify the exon 3 of DHH(NM_021044.3). Exon 3-F: 5’- TAGCGCGTGCAGCAGTCT-3’; exon 3-R: 5’- TGGTCTTGATTCAATCCTCCC-3’.

The PCR amplifications were performed as per the following procedure: an initial denaturation of 98 °C for 2 min, 10 cycles of denaturation at 98 °C for 10 s, annealing at 65 °C for 30 s, extension at 72 °C for 10 se c, then 25 cycles of denaturation at 98 °C for 10 s, annealing at 55 °C for 30 s, extension at 72 °C for 10 s and a final extension of 72 °C for 1 min. An ABI3730xl sequencer was used for sanger sequencing of the purified PCR products. The sequencing results were analyzed using the DNASTAR(Madison) software.

## Results

### Clinical manifestations

The two siblings were born at full term in Zhejiang Province, China. The pregnancies and perinatal periods of both patients were not uneventful. They had average height and weight for their age, according to the Chinese children’s standards. No significant disorders were found in the parents. The patients were evaluated by the DSD multidisciplinary team in the hospital, including paediatric urologists, paediatric endocrinologists, and geneticists.

#### Patient 1

**Fig. 1:**
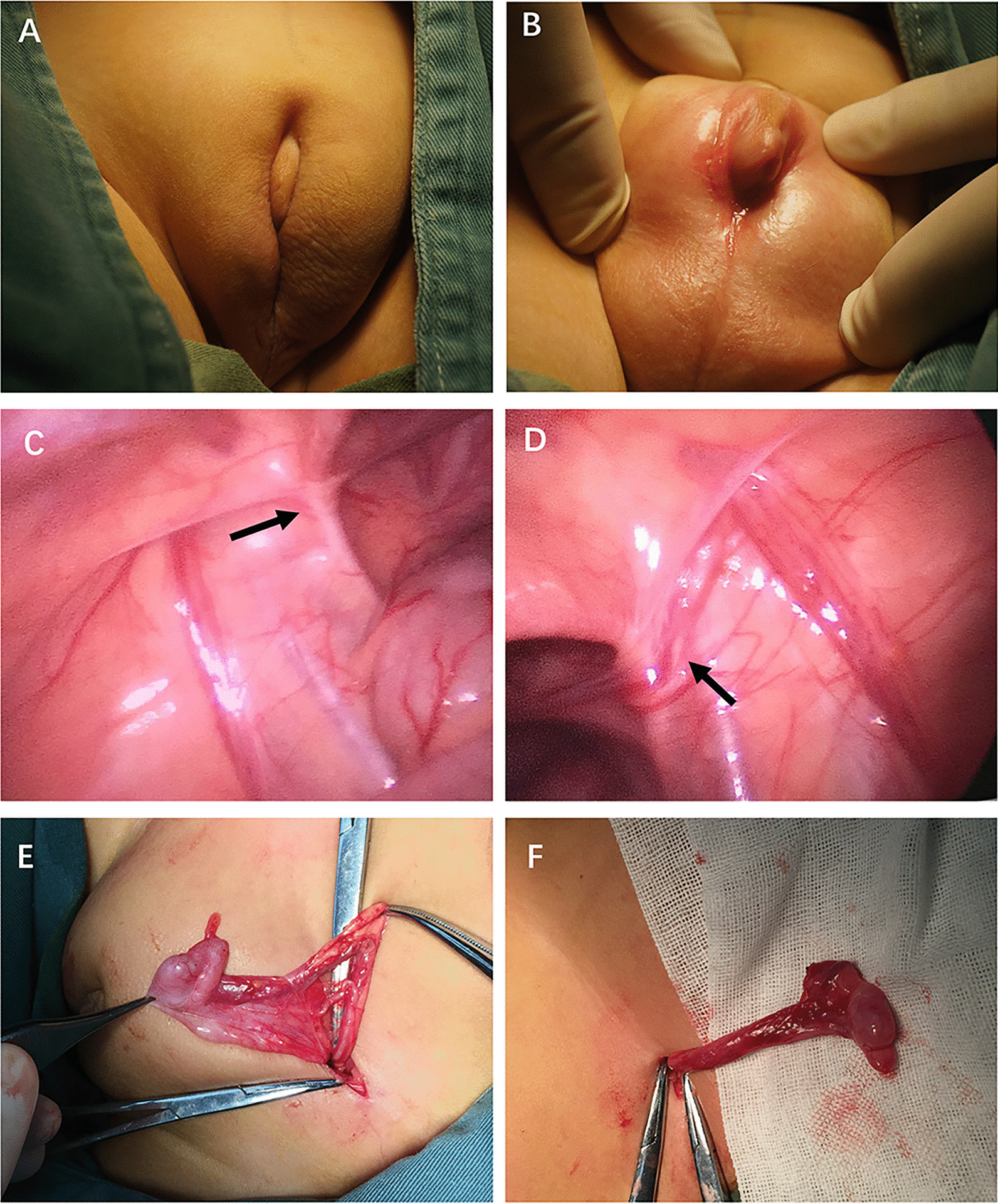
External and internal genitalia features of proband patient 1. **(A and B)** External genitalia appeared as a single perineal urogenital orifice and a mega-clitoris with almost complete scrotum fusion. **(C)** The left vas deferens (arrow). **(D)** The right vas deferens (arrow). **(E)** The left testis was in the left inguinal region. **(F)** The right testis was in the right inguinal region

The proband was the younger brother; he was 13-month-old. His social sex was female. His mother delivered him at 38 years old. He had a single perineal urogenital orifice and a mega-clitoris with almost complete scrotum fusion. His bilateral testes were palpable in the inguinal regions. He was assessed as stage III on the Prader scale and scored 5 on an external masculinization score (EMS) evaluative test (Fig. 1A and B). The karyotype was determined to be 46,XY, and the SRY gene was positive. The serum dihydrotestosterone level was lower than the normal range (17.48 pg/ml). The serum inhibin B concentration was 314.63 pg/ml. The 17-α hydroxyprogesterone concentration was 0.3 nmol/L. The AMH concentration was 103.37 ng/ml. The serum levels of FSH, LH, oestradiol, progesterone, prolactin, testosterone, HCG, adrenocorticotropin, and hydrocortisone were normal. The ultrasound test showed that two testes were in the inguinal regions. The volume of the left testis was 1.3 × 0.8 × 0.6 cm, and the volume of the right testis was 1.2 × 0.8 × 0.7 cm. His diagnosis was 46,XY DSD.

The patient underwent laparoscopic and cystoscopy explorations. The spermatic vessels and the deferent ducts were identified (Fig. 1C and D). The verumontanum was found. The investigation did not find a uterus, ovary, or vagina. The volumes of the bilateral testes were significantly smaller than average (Fig. 1E and F). However, the appearance of the testes and epididymis seemed normal. Bilateral gonadal biopsy was performed, and the pathological diagnosis confirmed that the bilateral gonads were testes. Finally, he underwent bilateral orchidopexy and two-stage hypospadias repair. The surgeries were performed successfully. During the 1-year follow-up, the appearance of the external genitalia was similar to that of normal males of his age; however, his penis was significantly shorter than that of normal males. Even so, the parents were extremely satisfied with the outcomes of the treatments.

#### Patient 2

The elder brother was a 16-year-old male who was also diagnosed with 46,XY DSD. His mother delivered him at 23 years old. He had had a single perineal urogenital orifice and a mega-clitoris with complete scrotum fusion. His bilateral testes were in the scrotums. He had been assessed as stage III on the Prader scale and scored 6 on the EMS evaluative test according to his medical history record. He had received the two-stage hypospadias repair surgeries in his childhood (aged 3 years old) in our hospital. Currently, the penis is smaller than the average range according to his age, and the urethral orifice is opened in the top of the glans. Two testes were palpable in the scrotums. The volumes of the testes were normal. The breasts were mildly developed as stage 2. The boy refused our photograph requests. His karyotype was identified as 46,XY, and the SRY gene was positive. The nerve conduction study results were normal. The ultrasound showed that the length of the penis was 7.6 cm. The volume of the left testicle was 3.9 × 2.0 × 1.9 cm, and the volume of the right testicle was 4.0 × 2.2 × 2.0 cm. The volume of the left breast was 1.8 × 1.4 × 0.7 cm, and the volume of the right breast was 2.0 × 1.9 × 0.6 cm.

### Genetic sequencing analysis

**Fig. 2:**
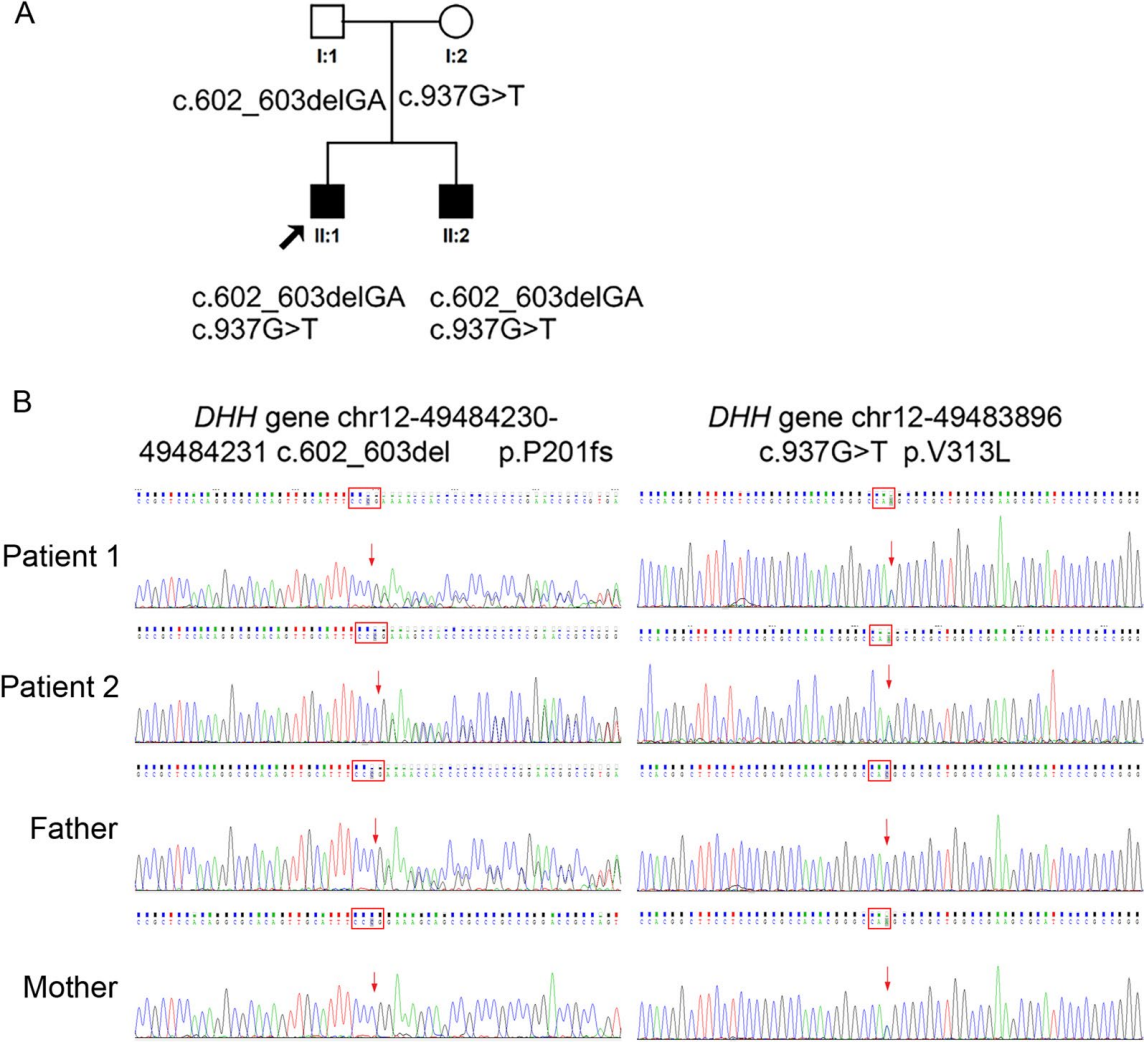
Genetic analysis of two siblings with 46,XY DSD. **(A)** Pedigree of the family. Squares indicate males, and circles indicate females. Dashed squares symbolize females or males affected with 46,XY DSD. The single line connects the two parents, who are not in a consanguineous marriage. The proband (II/1) was a 13-month-old male. His elder brother (II/2) is currently 16 years old but was 3 years old at the time of diagnosis. **(B)** Validation of the variants by Sanger sequencing of the parents and two patients

Next-generation sequencing was used to analyse the genes of the proband. The *SRY* gene was analysed in the two patients, and no mutations were found. Direct sequencing of *DHH* indicated a double heterozygous mutation in exon 3 (*DHH*; NM_021044.3) in both brothers. The c.602delC mutation was derived from their father, and the c.937G > T mutation was derived from their mother. The c.602delC mutation was a frameshift mutation that subsequently caused 136 amino acid changes. The c.937G > T mutation was a missense mutation that led to the change of a valine to a leucine at position 313 of the protein, according to the reference sequence NM_021044. The incidence is 1/16 for the siblings to share the same *DHH* mutation. No sequence variations were found in exon 1 or exon 2 of the gene. The next-generation sequencing results were verified by Sanger sequencing (Fig. 2A) using a pair of primers designed to amplify exon 3 of DHH.

All pathogenicity prediction algorithms (SIFT, PolyPhen2, and CADD) consider this mutation damaging or disease-causing. This variant is not present in the 1000 Genomes Project, dbSNP, or ExAC databases. Furthermore, we did not find any other mutation in DSD-related genes.

The pedigree of the family is shown in Fig. 2B.

## Discussion

DHH belongs to the hedgehog family, which consists of sonic hedgehog (SHH), DHH, and India hedgehog (IHH). They function as cell-to-cell signals and have embryonic roles in the specification of tissue patterning and cell differentiation [13]. The importance of *DHH* in male sexual development has been indicated in various studies. It has been reported that a homozygous mutation in the *DHH* gene can result in complete pure gonadal dysgenesis [10], partial gonadal dysgenesis [8], and even gonadal tumours [9] in patients with 46,XY. However, *DHH* gene mutations causing 46,XY have rarely been reported in the Chinese population [14, 15]. In a putative animal model study, DHH regulates testis formation, spermatogenesis, and the differentiation of peritubular myoid cells [16]. A missense mutation of the gene encoding DHH in mutant rats indicated that the *Dhh* gene might be essential for developing Leydig cells [17] and functions to secrete testosterone through a paracrine signalling mechanism. Furthermore, DHH is involved in the WT1-SOX8/SOX9-beta-CATENIN-DHH network in Sertoli cells, which is the signalling pathway for testis cord maintenance [18].

The DHH signalling pathway may ensure mammalian spermatogenic function and may be directly regulated by sex-determining pathways. DHH can be autocatalytically cleaved into the N-terminal portion and C-terminal portion. The N-terminal portion is soluble and contains the signalling activity [19]. The Hedgehog receptor Patched negatively regulates the activity and ciliary accumulation of Smoothened, a seven-transmembrane protein that is essential for transducing Hedgehog signalling [20]. DHH signalling is mediated by hedgehog coreceptors and cell adhesion molecules, and is downregulated by oncogenes (CDO and BOC). CDO and BOC bind all mammalian Hedgehog proteins in a conserved manner [9, 13]. Previous research has shown that BOC positively regulates hedgehog signalling [21]. The C-terminal portion is involved in precursor processing, which covalently attaches a cholesterol moiety to the N-terminal product, restricting the N-terminal product to the cell surface and preventing it from freely diffusing throughout the organism. In this study, the *DHH* frameshift mutation (c.602delC) resulted in changes to 136 amino acids, and the missense mutation (c.937G > T) led to the replacement of V with L at residue 313. These mutations may alter the normal functions of DHH by either restricting the processing or secretion of the N-terminal portion or by limiting its binding to its receptor Patched or coreceptors such as CDO and BOC. Thus, these changes would lead to incomplete sex development.

As we showed before, patient 1 was a 13-month-old male who was of female social sex, and patient 2 was a 16-year-old male. However, they had the same *DHH* gene mutations. The external genitalia of patient 1 were feminized, and two gonads were linguistic. In contrast, the external genitalia of patient 2 were penoscrotal hypospadias. The severity of DSD in these two patients with the same gene mutations indicates that male sex development may be associated with gene mutations and affected by penetrance of the gene, which may be due to pregnancy age or the mother’s living environment. The mother of both patients gave birth the younger brother when she was 38 years old, and the family moved from the rural area to the city.

Genetic analysis is a useful tool for doctors to determine the causes of diseases, make a specific diagnosis, and precisely treat an individual patient. In previous studies, most of the patients were diagnosed in adolescence or adulthood, and the chief complaint was amenorrhea, which meant that their social sex was female [22]. There has been no evidence to indicate their brain sex or the subsequent treatment after DSD diagnosis in these studies. However, an early diagnosis appears to be beneficial to the patient and family. Parents and paediatric urologists will have more time to discuss any issues and make better treatment choices to minimize harm to the children physically and psychologically.

## Conclusions

We identified novel compound heterozygous *DHH* mutations (c.602delC and c.937G > T) in two siblings diagnosed with 46,XY DSD. The c.602delC mutation caused changes to the subsequent 136 amino acids, and the c.937G > T mutation was a missense mutation that changed a valine residue to a leucine residue at position 313 of the protein. These two mutations suppress the normal functions of DHH, which affects the hedgehog signalling pathway.

## Data Availability

The raw sequence data reported in this paper have been deposited in NCBI-SRA with the BioProject accession number PRJNA855469.

## Abbreviations

DSD: Disorders of sex development
BOC: Brother of the cell adhesion molecule downregulated by oncogenes
SRY: Sex-determining region Y
SOX9: Sex-determining region Y-related high-mobility group box 9
MAP3K1: Mitogen-activated protein kinase kinase kinase 1
NR5A1: Nuclear receptor subfamily 5 group A member 1
WT1: Wilms' tumour gene 1
EMS: External masculinization score
AMH: Anti-mullerian hormone
FSH: Follicle-stimulating hormone
LH: Luteinizing Hormone
HCG: Human chorionic gonadotropin
SHH: Sonic hedgehog
IHH: India hedgehog
CDO: Cell adhesion molecule downregulated by oncogenes
